# Intersectional Structural Stigma, Community Priorities, and Opportunities for Transgender Health Equity: Findings from TRANSforming the Carolinas

**DOI:** 10.1017/jme.2022.86

**Published:** 2022

**Authors:** Tonia Poteat, Ames Simmons

**Affiliations:** 1: UNIVERSITY OF NORTH CAROLINA-CHAPEL HILL, CHAPEL HILL, NC, USA; 2: DUKE UNIVERSITY, DURHAM, NC, USA.

**Keywords:** Transgender, Health Equity, HIV, Community Engagement

## Abstract

In this manuscript, “Intersectional Structural Stigma, Community Priorities, and Opportunities for Transgender Health Equity,” Poteat and Simmons outline the legal and policy barriers that impede efforts to end the HIV epidemic among transgender people in the South. They present qualitative and quantitative data from a community engaged research study conducted with transgender adults and other key stakeholders as well as finding from an analysis of policies impacting transgender people in both states. Violence prevention and decriminalization are highlighted as key policy initiatives that would advance health equity for transgender people.

## Background

Transgender and gender nonbinary (trans) individuals — defined as people whose gender differs from their sex assigned at birth — experience significant health inequities. A growing number of studies have identified disparities in access to health care, mental and physical health, and a variety of specific health conditions, including HIV.^1^ Nationwide, an estimated 9% of trans people are living with HIV compared with 0.3% of the general U.S. population. Estimates by gender indicate that 14% of trans women and 3% of trans men are living with HIV, with Black and Latine populations bearing the brunt of the epidemic. An estimated 44% of Black trans women and 26% of trans Latinas in the U.S. are living with HIV.^2^ The majority of Black and Latine transgender people with HIV reside in the South, a geographic and cultural region defined by the U.S. Census bureau as Alabama, Arkansas, Delaware, the District of Columbia, Florida, Georgia, Kentucky, Louisiana, Maryland, Mississippi, North Carolina, Oklahoma, South Carolina, Tennessee, Texas, Virginia, and West Virginia.^3^

This article intends to show that these racialized and gendered health inequities are related to interwoven precarity across the lives of transgender people, not only in the delivery of healthcare, but also including discrimination in employment, housing, and public spaces and services. The impact of these precarious conditions is compounded for Black, Latine, and other people of color due to structural factors such as laws and policies that ultimately increase HIV vulnerabilities.

This article intends to show that these racialized and gendered health inequities are related to interwoven precarity across the lives of transgender people, not only in the delivery of healthcare, but also including discrimination in employment, housing, and public spaces and services. The impact of these precarious conditions is compounded for Black, Latine, and other people of color due to structural factors such as laws and policies that ultimately increase HIV vulnerabilities.

Employment discrimination limits income generation opportunities for many trans people. For example, 15% of transgender NC respondents^4^ and 10% of transgender SC respondents^5^ in the 2015 U.S. Transgender Survey (USTS) reported being unemployed, while the U.S. unemployment rate at that time was 5%. Relatedly, 29% of transgender North Carolinians reported living at the poverty level in 2015, compared to the U.S. poverty rate of 14% at the time of the survey.^6^ Likewise, 28% percent of transgender South Carolinians reported living at the poverty level in 2015.^7^ Limited income generation often results in high rates of survival sex work. Trans people are often constrained to street-based sex work where the ability to negotiate condom use is limited and exposure to the criminal legal system is high.

Lack of predictable income makes secure housing harder to obtain for transgender people. Almost one-third of trans North Carolinians in the 2015 USTS reported experiencing homelessness at some point in their lives,^8^ and over one-third of trans South Carolinians had experienced homelessness during their lives.^9^ Houselessness often means spending more time in public places and spaces. For example, 16% of trans North Carolinians reported verbal harassment in public accommodations.^10^ Even more startling, in SC, 24% of trans people surveyed reported verbal harassment in public accommodations, and 1% reported physical assault in public accommodations.^11^

Widespread healthcare discrimination creates barriers to engagement in care, including HIV prevention and treatment. Discrimination is particularly problematic for trans people in the southern U.S. where few legal protections exist and denial of healthcare services for trans people is more common than in other regions.^12^

In the Transgender Law Center’s recent Southern Trans Report, 44% of trans participants identified primary and HIV-inclusive health care as the top issue impacting the trans community.^13^ Of the 233 trans South Carolinians who participated in the 2015 USTS, 37% of individuals who had seen a health care provider in the prior year reported at least one negative experience related to trans stigmatization, including refusal of treatment, verbal harassment, assault, or having to teach the provider about transgender people in order to get appropriate care.^14^ In the prior year, 22% of participants did not seek healthcare when needed due to fear of mistreatment.^15^ Data are similar for the 686 transgender North Carolinians surveyed: 29% reported at least one negative experience in the previous year, and 26% did not seek care due to fear of mistreatment.^16^ The particularly heavy burden of HIV borne by Black and Latine trans people in the South as well as the challenging sociostructural environment for trans individuals in the southern U.S. necessitates a deeper and broader understanding of the barriers in access to gender-affirming care as well as opportunities to inform change that will improve the health of trans people in the region.

## Introduction to TRANSforming the Carolinas Project

TRANSforming the Carolinas was a research project led by investigators at the University of North Carolina — Chapel Hill (UNC), the University of North Carolina — Charlotte (UNCC), and the University of South Carolina (USC). The National Institutes of Health (NIH) funded the study via a supplement to the UNC Center for AIDS Research as part of the federal “Ending the HIV Epidemic: A Plan for America (EHE),” a ten-year initiative to reduce new HIV infections by 90 percent by 2030. The objective of TRANSforming the Carolinas was to identify regionally relevant, structural, social, and individual drivers of disparities in health care access — particularly HIV prevention and care — among trans people of color in North Carolina (NC) and South Carolina (SC). Findings from the study are intended to inform policy and practice interventions to improve healthcare for transgender people of color by addressing their self-identified needs.^17^

Study investigators used four methods to identify and prioritize potential strategies to address HIV among trans people in NC and SC: individual key informant interviews, an online survey, policy analysis, and focus groups. The 12 key informant interviews were held via videoconference with medical providers, social service providers, public health officials, and policy makers in NC and SC. During the interviews, participants described existing barriers and challenges to engaging trans people of color in health and social services as well as potential strategies to improve engagement. Trans adults aged 18 and older in NC and SC (N=124) consented to participate in the self-administered survey; and 101 completed all questions. The survey assessed HIV risk and testing behaviors, barriers to and facilitators of engagement in HIV prevention and treatment services, experiences of stigma and discrimination, access to gender-affirming care, and community priorities for intervention. Four focus groups with a total of 12 individuals were held via videoconference with trans community members in NC and SC. During the group discussions, we conducted a community asset mapping exercise to facilitate identification of available resources to address barriers to HIV prevention and care identified during the survey and key informant interviews. The policy analysis used a standardized search and abstraction form to develop an inventory of laws and policies in NC and SC that pose barriers to fully engaging transgender people of color in HIV prevention and care services. The review and analysis were not limited to HIV laws but included any laws and policies that may increase stigma, impact access to gender affirmation, or disproportionately impact gender minorities and ethnic and racial minorities.

## Highlights from the Survey Results

The average age of participants was 32 years with a range from 18–81 years of age. Gender identities included 39% trans men, 31% trans women, 25% nonbinary, and 5% who preferred not to answer. 36% of respondents identified as White, 31% as Black, 20% as multiracial; 14% reported Latine ethnicity, regardless of racial identity. Of the 83 participants who reported having an HIV test, 11% reported testing positive. All of the participants with HIV were Black (89%) and/or Latine (11%). More than half of all participants reported experiencing some type of violence related to their gender identity. Six out of every 10 respondents had been homeless at some point in their lives. Less than one third were employed full-time; 8% were full-time students. A remarkable 51% reported a current income below the federal poverty level. Sixty percent reported having no identity documents that listed the correct gender. Of the 81 participants with health insurance, 52% reported that their insurance covered gender-affirming hormone therapy, but only 19% reported that gender-affirming surgery was covered by their insurance. Sixty-five percent had attempted to get gender-affirming care in the prior 12 months; of those, 72% were successful in getting care.

## Highlights from the Policy Analysis

The policy research phase of TRANSforming the Carolinas consisted of an assessment of laws and policy that may create barriers to ending the HIV epidemic for transgender people of color in NC and SC. This report provides a high-level assessment of policy at the state and municipal levels in both states, taking a broad, systems-level approach.

At the municipal level, this policy analysis only looked at the city of Charlotte in NC, because Charlotte was the only jurisdiction in NC that received a grant for Ending the HIV Epidemic. Although the entire state of SC received a grant for Ending the HIV Epidemic, for purposes of this project, the SC cities whose policies were analyzed included Charleston and Columbia. Other municipalities in SC have enacted ordinances protecting sexual orientation and gender identity that were not included in this analysis.

This phase of the research did not attempt to capture the lived experience of trans people in NC and SC, which was the focus of later phases of the research project described elsewhere in this article. Before diving into the results of the policy research, we first defined who are transgender people of color in NC and SC.

An estimated 29,800 trans people of color live in NC. This number was derived by adding the Williams Institute’s June 2022 population estimates of 15,700 Black trans North Carolinians; 2,100 Asian trans North Carolinians; 8,200 Latine trans North Carolinians; and 3,800 trans North Carolinians of all other race/ethnicity groups except White.^18^ Approximately 0.87% of NC’s adult population identifies as trans.^19^ However, this figure is likely underreported as there are likely many more trans people in NC who do not openly identify as trans due to risks of harassment, intimidation, and violence.

An estimated 7,600 trans people of color live in SC. This number was derived by adding the Williams Institute’s June 2022 population estimates of 5,400 Black trans South Carolinians; 300 Asian trans South Carolinians; 1,300 Latine trans South Carolinians; and 3,800 trans South Carolinians of all other race/ethnicity groups except White.^20^ Approximately 0.47% of SC’s adult population identifies as trans.^21^ Similar to NC, this figure is likely underreported due to fear of harassment, intimidation, and violence as an openly trans person.

To broadly summarize the policy research, both NC and SC have significant legal and policy barriers to ending the HIV epidemic for trans people that are described below. In order to illustrate these conclusions, we present an analysis of the civil rights policy generally in each state; each state’s criminalization of identities such as living with HIV, using syringes, and engaging in sex work; and the health policy that makes it more difficult for trans people of color to get care. Finally, because some conditions of living known as social determinants of health create health disparities,^22^ we detail below each state’s policy postures with respect to rights to employment, housing, public places and spaces, and freedom from violence. Taken as a whole, NC has a slightly better law and policy environment for trans people than SC. This is not unexpected given South Carolina’s closer proximity to the deep South where anti-Blackness is even further entrenched in structures like law and policy.

Looking at the civil rights environment of each state at a high level, there are no statewide protections against discrimination based on many identities commonly held by trans people. Neither state has prohibited discrimination on the basis of sexual orientation, gender identity, HIV status or disability, or gender dysphoria disability, although there are certain pockets of protections. For example, NC prohibits discrimination based on HIV status in some aspects of employment,^23^ but does not consider HIV or gender dysphoria to be disabling conditions under the Persons with Disabilities Act.^24^

Sometimes residents can gain protections at the municipal level that are not available statewide. In states that have majorities of conservative lawmakers, it can be prohibitively difficult to pass statewide nondiscrimination laws like those described above, but sometimes cities or counties can pass ordinances prohibiting discrimination. Prior to December 1, 2020, when a key NC law expired, SC cities had enacted more protections than NC cities. NC Session Law 2017-4, also known as HB142, prohibited local governments from regulating private employment or public accommodations until December 1, 2020, after which some municipalities in NC passed ordinances protecting residents from discrimination based on sexual orientation and gender identity.^25^

Another important policy area we examined was the criminalization of identities, in other words, criminal penalties associated with living with HIV, using syringes, or engaging in sex work. Both states have criminal laws based on HIV status. NC’s law is relatively more modernized because it accounts for scientific consensus that undetectable viral loads make HIV non-transmissible, and that effective pre-exposure prophylaxis can prevent transmission of HIV.^26^ NC’s law makes violation of the HIV control measures a misdemeanor and does not include engaging in sex work while living with HIV, whereas SC’s law is a felony and does include sex work.^27^ Both states include shared needle use^28^ in their HIV criminalization but NC has a legalized syringe exchange program that may make access to syringes easier.^29^

Any criminalized identity makes someone more vulnerable to intimate partner violence because it creates an opportunity for an aggressor to exert power and control by threatening to report the survivor to authorities.^30^ Trans people living with HIV may have multiple criminalized identities because they may share syringes for hormones or silicone injections if they don’t have access to medical sources, and because some trans people exchange sex for money, goods, or services.

There is an emerging public health strategy known as molecular surveillance that is increasing concern about HIV criminalization. Molecular data analysis is a process by which health departments repurpose the genetic sequences that healthcare providers previously obtained from patients to check for HIV drug resistance, in order to identify similar sequences in clusters.^31^ The advent of molecular surveillance as a public health tool may create additional pathways to criminalization, as needle-sharing mutual aid webs and sex worker client networks may be revealed without the knowledge or consent of the people living with HIV.^32^

It is difficult to know how many trans people might be arrested or convicted because of their HIV status in either state. At the time of this analysis, neither state consistently reported HIV data disaggregated by gender identity, so examination of arrest and conviction records does not provide any insight as to how many trans people were arrested or convicted of violating HIV control measures and laws. Also, sometimes HIV criminalization is not a product of violating HIV control measures, but rather results from violation of some other criminal law. Some people living with HIV who face other criminal charges may also face HIV penalty enhancements for those violations at the discretion of the prosecutor. Therefore it is challenging to get a true picture of the impact of HIV criminalization without examining every single criminal arrest and conviction under any law to check for penalty enhancements.

Once trans people are arrested, they can face harassment and violence from carceral systems because of being trans. While both NC^33^ and SC^34^ maintain corrections policies that provide routes for trans people to obtain gender-affirming services while in custody, it is beyond the scope of this research to establish the extent to which the policies are being followed. However, both NC and SC carceral systems are bound by a 2022 opinion in the Fourth Circuit Court of Appeals holding that trans people with gender dysphoria who are housed according to their genitalia and denied access to medically necessary hormone treatment plausibly may plausibly state claims of gross negligence and violation of the Americans with Disabilities Act.^35^

Our third area of policy analysis concerned laws and regulations that pose barriers to effective healthcare. For example, neither NC nor SC has expanded Medicaid. Since trans people are disproportionately likely to be low-income, failing to expand Medicaid means some trans people do not have access to healthcare at all outside of community clinics. The positive news is that neither state prohibits insurance coverage of transition-related healthcare. However, neither state prohibits *exclusions* of transition-related healthcare either. Furthermore, neither state affirmatively requires coverage of transition-related healthcare. Even if transgender people have insurance, they may not be able to get medically necessary care to begin or maintain gender-affirming treatment.

Finally, we explored several environmental factors that have been shown to create health disparities. These social determinants of health that were researched in NC and SC include employment, housing, public places and spaces (called “public accommodations” in policy), and hate violence.

There are no statewide protections against discrimination by private employers in either state. However, if a trans person works for the state in NC^36^ or for the cities of Charlotte,^37^ Charleston,^38^ or Columbia,^39^ they cannot be fired because of gender identity. A newly-enacted revision to the Charlotte City Code effective January 1, 2022, prohibits discrimination by private employers within the city.^40^ Since employment is the primary way people in the U.S. gain health insurance, and since trans people commonly encounter employment discrimination as described above, except for the few pockets of municipal protections, the prevalent lack of employment nondiscrimination laws may keep trans people from being able to access healthcare.

There are no protections against discrimination based on gender identity in housing except in Charleston^41^ and Columbia^42^ in SC. Without stable housing, it is difficult for trans people to maintain employment and to safely store medications.

Residents of Charleston,^43^ Columbia,^44^ and Charlotte^45^ are protected against discrimination on the basis of gender identity in accessing public spaces. However, NC cities are permanently prohibited from protecting trans people’s access to facilities.^46^ While cities cannot affirmatively protect trans people’s access to multiple occupancy restrooms, showers or changing rooms, a federal court has approved a consent decree binding NC Governor Roy Cooper from applying this provision in a way “that bars, prohibits, blocks, deters, or impedes trans people from using public facilities in accordance with their gender identity or subjects trans people to arrest, prosecution, or criminal sanctions for doing so.”^47^ Since trans people disproportionately face harassment and discrimination in public spaces as described above, including hospitals and healthcare centers, it is more difficult for them to participate in public life.

The only locations that have laws against hate violence based on gender identity are SC cities of Charleston^48^ and Columbia.^49^ The epidemic of lethal hate violence against trans women threatens the health and well-being of all trans people in NC and SC. Over the past seven years, nine trans North Carolinians have been the victims of lethal hate violence: Elisha Walker (Aug. 13, 2015), Sherrell Faulkner (May 16, 2017), Derricka Banner (Sep. 12, 2017), Chanel Scurlock (June 6, 2019), Bubba Walker (July 2019), Monika Diamond (Mar. 18, 2020), Jenna Franks (Feb. 24, 2021), Jaida Peterson (Apr. 4, 2021), Remy Fennell (Apr. 15, 2021), and Sasha Mason (May 13, 2022). Six trans South Carolinians have had a similar fate in the past three years: Sasha Wall (April 1, 2018), Regina Denise Brown (October 7, 2018), Denali Berries Stuckey (July 20, 2019), Pebbles LaDime Doe (August 4, 2019), Thomas Hardin (May 2, 2021), and Marquiisha Lawrence (Nov. 2, 2021).^50^

**Figure 1:**
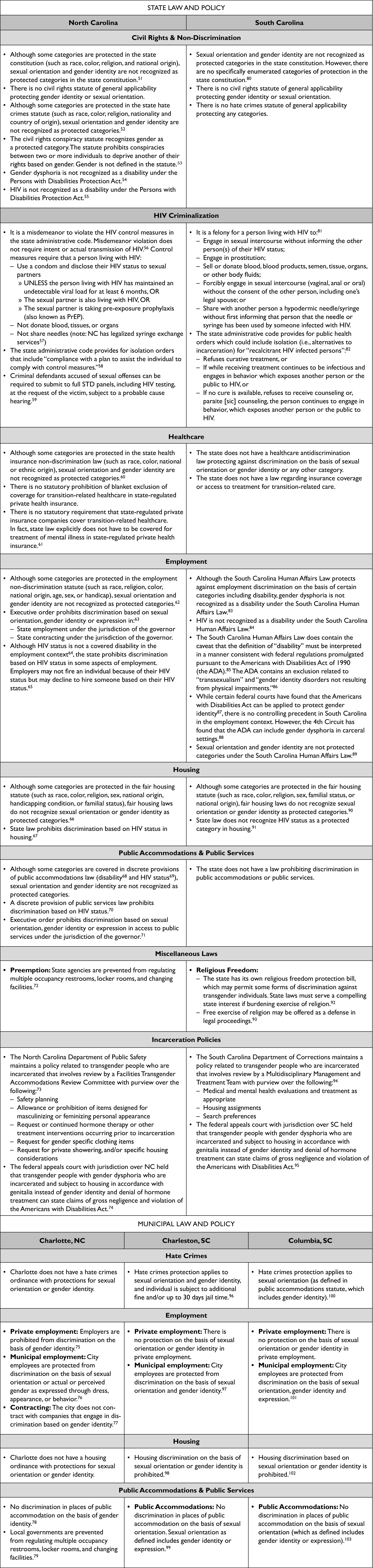
Intersectional Structural Stigma, Community Priorities, and Opportunities for Transgender Health Equity

To see a detailed table of the provisions of policy that affect trans people with HIV, please see the appended tables (Figure 1) divided between state policy and local policy.

## Community Priorities (from survey) and Community Assets (summary of focus group results and key informant interviews)

The top five priorities selected by survey participants included 1. preventing violence, harassment, and bullying, 2. access to safe, affordable housing, 3. insurance coverage for gender care, 4. access to gender care, and 5. making it easier to change gender on identity documents. These priorities did not vary significantly by race or ethnicity. Other highly ranked priorities included: preventing police violence, access to employment, and education of healthcare providers about trans health. Community assets identified during the key informant interviews and focus groups included local trans-led organizations and specific healthcare providers known to be gender-affirming. It was notable during the focus groups that while participants did not know each other, those who knew of resources typically identified the same local resources. Participants also sought out connections with one another, some exchanging information to remain in contact. This highlights ways in which trans individuals may be socially isolated from one another, especially during pandemic restrictions that precluded social gatherings.

## Discussion of Opportunities to Advance Health Equity for Trans People via Policy Change

Trans people in NC and SC face a number of policy barriers that must be addressed in order to end the HIV epidemic. Additional research, including the use of mathematical modeling that helps us make projections where data are missing, may help fill in gaps in data and provide policymakers with additional evidence for policy reform that would improve health equity for trans North Carolinians and South Carolinians living with HIV. Specifically, we recommend that the research community prioritize to answer the following questions:

- **Violence Prevention**
  - How much morbidity is caused by violence against trans people living with HIV?
  - Without intervention, how many trans people living with HIV will face violence over the next five years?
  - What are the most cost-effective interventions to prevent interpersonal violence in the lives of trans people living with HIV?
  - What are the appropriate metrics to capture the number of incidents of interpersonal violence among trans people living with HIV that were prevented by any given intervention?
- **Anti-Criminalization**
  - What impact would repealing laws that criminalize the failure to disclose HIV status have on the incidence of HIV among trans people?
  - What role does systemic racism play in the way trans people of color interact with the criminal legal system under these laws?
  - If HIV criminalization laws are not repealed, how many trans people will avoid getting tested for HIV so that they will not be charged with a crime for failing to disclose their status to their partners?
  - What is the minimum amount of funding for HIV decriminalization advocacy campaigns that is required to accomplish repeal during one state legislative session?
  - Across the entire country, which communities are the top priority for decriminalization, based on potential impact of decriminalization?

Policy is one of many hurdles that present barriers to effectively ending the epidemic of HIV for trans people of color in NC and SC. The policy environment in state legislatures in NC and SC and across the country is not one that is bending toward advancing health equity for trans people. NC’s trans communities remain deeply scarred by the 2016 adoption of a policy also known as HB2 that restricted access to public restrooms and changing rooms based on the sex shown on a person’s birth certificate.^104^ This law insidiously also undermined rights against employment discrimination^105^ and restricted cities’ and counties’ ability to advance better policy at the local level.^106^ While the birth certificate requirement for restroom access was repealed the following year as mentioned above, NC cities and counties remain permanently prohibited from affirmatively protecting the access of trans residents and visitors to public facilities.^107^

HB2 and HB142 have had a demonstratively negative impact on health outcomes for NC’s trans community: an estimated 31% of trans and gender non-conforming North Carolinians experienced discrimination after the passage of HB2, 14% delayed getting healthcare they needed, and 5% experienced increased violence.^108^ Two out of every three respondents reported increased anxiety, and one in two reported an increase in depressive thoughts, with as many as 5,000 people considering suicide.^109^

In the years since HB2 and HB142 passed, a number of bills have been proposed in both NC and SC that would cause increasing levels of harm to trans people. For example, both NC^110^ and SC^111^ have seen the introduction of legislation that would restrict the ability of trans youth to participate in sports consistent with their gender identity. Both NC^112^ and SC^113^ have also seen legislation introduced that would restrict the ability of youth to access medically necessary gender-affirming healthcare.

Youth-serving organizations like the Trevor Project have highlighted the impact that this restrictive policy environment has on trans and gender non-conforming youth. For example, polling released in January 2022 showed that recent attention to laws restricting the rights of trans people has negatively impacted their health, with 85% of trans and non-binary respondents reporting negative impacts, and 37% percent of trans and non-binary respondents reporting that the debates have very negatively impacted their mental health.^114^

However, the inverse is also true: policy that affirms trans people’s identities not only does not have a negative impact on their health, but shows promise for increased positive outcomes. For example, state-level policy that would prohibit private insurers from discriminating on the basis of gender identity has been associated with decreased or no change in suicidality among gender minority people.^115^ In other words, restrictive state policies affecting trans people were associated with victimization and discrimination and lifetime number of suicide attempts, whereas states with affirming policies were less likely to be associated with minority stressors.^116^

Positive, affirming policy that is based on deep relationships with community-based organizations presents the best opportunity to make meaningful change to end the HIV epidemic for trans people of color in NC and SC. We recommend that policymakers in NC and SC work with community groups to craft policy that removes barriers to good health such as those we have detailed in this article. Additionally, community groups in NC and SC who are not already advocating for these policies should examine the list we have compiled for any that fit their mission, and consider adding other policies that would be impactful that we did not include. If the research community is not already connected to community groups, they should build those relationships and undertake, with the participation of the community, the research we recommend regarding violence prevention and anti-criminalization. Other states and even federal lawmakers should examine their policy landscapes and broaden their agendas to include the areas we have highlighted here.

## Note

Dr. Poteat reports grants from National Institutes of Health, during the conduct of the study, other from ViiV Healthcare, and other from Merck & Co., outside the submitted work. Mr. Simmons has no conflicts to disclose.
